# The primacy of social over visual perspective-taking

**DOI:** 10.3389/fnhum.2013.00558

**Published:** 2013-09-10

**Authors:** Henrike Moll, Derya Kadipasaoglu

**Affiliations:** Department of Psychology, University of Southern CaliforniaLos Angeles, CA, USA

**Keywords:** referential communication, perspective-taking, joint attention, theory of mind (ToM), intersubjectivity

## Abstract

In this article, we argue for the developmental primacy of social over visual perspective-taking. In our terminology, social perspective-taking involves some understanding of another person's preferences, goals, intentions etc. which can be discerned from temporally extended interactions, including dialog. As is evidenced by their successful performance on various reference disambiguation tasks, infants in their second year of life first begin to develop such skills. They can, for example, determine which of two or more objects another is referring to based on previously expressed preferences or the distinct quality with which these objects were jointly explored. The pattern of findings from developmental research further indicates that this ability emerges sooner than analogous forms of visual perspective-taking. Our explanatory account of this developmental sequence highlights the primary importance of joint attention and the formation of common ground with others. Before children can develop an awareness of what exactly is seen or how an object appears from a particular viewpoint, they must learn to share attention and build common “experiential” ground. Learning about others' as well as one's own “snapshot” perspectives in a literal, i.e., optical sense of the term, is a secondary step that affords an abstraction from all (prior) pragmatic involvement with objects.

Visual perspective-taking tasks typically entail another agent who embodies the spatial coordinates that the participant has to consider. They are thus at least minimally social in the sense that someone else is co-present and available for social interaction (see Schütz, 1932). In line with this, children with autism, whose difficulties are known to be first and foremost social in nature, struggle to detemine how others see things from their viewpoint (Reed, 2002; Hamilton et al., 2009; Yu et al., 2011; but see Hobson, 1984). At the same time, however, a “cold-cognitive” assessment or computation of how objects relate to one another in space is arguably less of a social affair than understanding another's affective, conceptual, or epistemic attitude toward a situation (see Fishbein et al., 1972).

In this article, we adopt the opposition of visuo-spatial and social perspective-taking employed by the editors. We will argue from a developmental approach that social perspective-taking is primary and precedes visual perspective-taking in human ontogeny. Our claim is that children first learn to take perspectives in situations that are not defined by differences in how self and other perceive objects visually but by differences in their experiential backgrounds, i.e., in what they did, witnessed, or heard. It might seem more complex to keep track of another's prior encounters and engagement with things than to compute his instantaneous visuo-spatial relation to an object in the room. Yet, it will become clear that infants readily note and update “experiential records” (Perner and Roessler, 2012, p. 522). Registering and remembering what others did, witnessed, or mentioned is less of a task demand for them than a helpful cue to others' goals and intentions. Per definition, no such cues from prior encounters are available in visual perspective-taking tasks that revolve entirely around momentary visuo-spatial relations.

First, we will review referential ambiguity tasks that are typically solved in the second year of life. It will become obvious that infants readily rely on others' previous expressions of attitude, their prior attentional engagements with objects, and previous discourse to solve the reference problem. These manifold abilities of infants to establish reference against the background of prior interactions are subsumed under “social perspective-taking.”

An overview of studies on visual perspective-taking will show that this ability has its onset noticably later. Again, it is generally taken for granted that perceptual perspective-taking precedes and serves as a foundation for the “deeper” forms of social perspective-taking (Kessler and Thomson, 2010). The same is suggested by accounts of mutual knowledge according to which physical co-presence is the easiest and least error-prone way to arrive at mutual knowledge (Clark and Marshall, 1981; Schiffer, 1972). These assumptions are seriously called into question by the empirical fact that visual perspective-taking does not precede but follows social perspective-taking ontogenetically.

An excursion into the early development of graphic skills lends further support to the idea that knowledge of visual perspectives is a relatively late cognitive achievement that is derivative of social perspective-taking. We will conclude with a programmatic attempt to explain this developmental sequence with the social and cooperative nature that sets humans apart from other animals.

## The role of the experiential background

### Prior affective expressions

Affective displays are key indicators as to how people will behave toward objects. In a seminal study, 14− and 18-month-old infants were presented with two food items: crackers and broccoli (Repacholi and Gopnik, 1997). The infants opted for the crackers, whereas an adult displayed the opposite preference. When the adult, without looking at either dish, later requested food from the infants, the younger ones gave her what they themselves liked (crackers), whereas the older ones selected the broccoli.

As was made clear by Perner et al. (2005), understanding perspectives in sensu strictu, as evidenced by an explicit acknowledgment of different takes on the self-same thing (“I cannot stand broccoli, but she likes it!”) is not necessary for this test. The infants just had to realize that the other and broccoli “go together,” and so an understanding of objective “person-object couplings” suffices to pass this test. Nonetheless, the older infants were able to learn about the other's taste preference from her prior expressions. A study by Egyed et al. (2007) confirmed that, in the absence of ostensive cues (which gear infants toward more object-centered interpretations such as “Broccoli is good”; see Gergely et al., 2007), 14-month-olds track specific persons' affective displays toward objects and expect them to behave in accordance with them later.

“Emotional eavesdropping” (Repacholi and Meltzoff, 2007) provides further support that infants act differently vis-à-vis others depending on their previously expressed affective attitudes. When 18-month-olds witness an adult reprimand another for performing a novel action, the infants later imitated the act less when the adult was present as opposed to when he was absent. Independently of their own desire or interest to perform an act, infants thus alter their behavior as a function of others' attitudes toward objects and actions.

### Prior engagement

Infants use various other cues to disambiguate reference. A powerful one is the other's familiarity with or ignorance of objects and their locations (see O'Neill, 1996, for an influential study with 2− and 2–5-year-olds). In their modification of a word learning study (Akhtar et al., 1996), Tomasello and Haberl (2003) found that 1-year-olds knew which of three objects an adult requested from them based on her prior engagement with the objects. When the adult excitedly asked infants for a toy, 12-month-olds chose the one that was new for the adult because she failed to see it in the past. Even though the infants themselves were equally familiar with all toys, their responses showed that they knew what the other had and had not witnessed a few moments prior.

MacPherson and Moore (2010) directly contrasted what an adult knew with what the infant herself was familiar with. In their study, two objects were mutually familiar for adult and infant, a third object was new for the infant and a fourth was new for the adult, but “old” for the infant. When the adult later excitedly requested a toy, 13-month-olds egocentrically chose what was new for themselves, while 19-month-olds selected the toy that was new for the adult and not for them.

Many studies have not just confirmed that infants readily track others' experiential backgrounds but have also yielded insights into the scope and limits of this skill. Joint attention has been shown to play an important role in interactive test situations. Having observed as mere onlookers how an agent engages with objects was insufficient for 14-month-olds to identify what the agent requested from them based on his knowledge vs. ignorance of the different objects. When the infant and the agent jointly engaged around the objects, infants successfully determined which things the other did and did not know (Moll and Tomasello, 2007; Moll et al., 2007, 2008). In contrast to mere onlooking, joint attention makes the co-attenders' familiarity with the object “mutually transparent” (Eilan, 2005)—it leaves no room for doubt that the object has been registered. Furthermore, it seems that unless the questioner clearly conveys that her excitement is elicited by something that is new for her individually, infants have a general bias to point out what is mutually familiar and unifies self and other in prior bouts of shared experiences (Saylor and Ganea, 2007; Liebal et al., 2009, 2011).

In a study that aimed to test false belief understanding, infants saw an adult as striving for different goals depending on what he witnessed earlier (Buttelmann et al., 2009). After the adult had placed an object in a box, he either attentively watched it being moved to a different container (true belief) or failed to witness the transfer (false belief). When the adult later approached the box from which the object had been removed, 18-month-olds helped him to get the box open (“He must want something else from this box!”) in the true belief, but retrieved the object from its new location in the false belief condition. Again, this demonstrates that infants take what others have witnessed into account when acting and responding toward them. They revert to the background constituted by past experiences and use it to inform them about an agent's desires, goals, and intentions.

Looking-time studies on false belief understanding further support this idea (e.g., Onishi and Baillargeon, 2005; Surian et al., 2007; Kovács et al., 2010). Even in their first year of life, infants look longer when they see an agent acting in a way that disaccords with his prior perceptual experiences (he acts as if he knows something he did not observe) than when they see him behave in ways that are consistent with what he observed. Whether belief understanding can be captured with this method remains the subject of an ongoing debate (Perner and Ruffman, 2005; Low and Perner, 2012), but what this research unequivocally demonstrates is that infants at a very young age are aware of what others have and have not registered perceptually. The findings also relativize the importance of joint attention suggested by interactive studies, because infants in looking-time tests usually do not jointly attend with the other and, in some cases, have not even reached the age at which they are able to do so. Joint attention might thus only play a critical role when infants have to directly respond to the agent in a communicative or cooperative act, which might require a more explicit understanding of knowledge and ignorance.

### Prior discourse

To not talk past, but speak with each other, interlocutors must know what they can and cannot presuppose as mutually given. Part of what defines the mutually given is the shared prior discourse—what Clark and Marshall (1981) refer to as “linguistic co-presence” in their model of mutual knowledge formation. Anecdotal evidence of “egocentric speech” (Piaget, 1929, 1955) alongside experimental data questioned children's skills in communicative perspective-taking. Young children tend to use pronouns (e.g., O'Neill and Holmes, 2002) and definite articles (Maratsos, 1976; Power and dal Martello, 1986) without having provided the antecedent. Their descriptions are often not specific enough to allow the listener to discern reference—even after requests for clarification were made, thus challenging effortless communication (see Glucksberg and Krauss, 1967; Deutsch and Pechmann, 1982; Sonnenschein and Whitehurst, 1984). Generally, young children have a tendency to underestimate the informativeness that is needed to communicate effectively (Olson and Torrance, 1987).

At the same time, evidence accumulates that even infants adjust their (speech) behavior according to what has been shared linguistically. For example, 2-year-olds use more informative naming constructions when a referent is new than when it is given, in the sense that it was part of previous discourse. Matthews et al. (2006) had 2-year-olds watch a video of a character performing an action (e.g., a clown jumping) together with an assistant. The assistant mentioned the character to the child. Another adult, who had either participated in the discourse or not then asked children to narrate what happened. In their replies, the children referred to the character more often with a pronoun (instead of a full noun) when their interlocutor had participated in the prior discourse than when he was not part of this discourse (see Nayer and Graham, 2006, for similar results with 3-year-olds). In Clark and Marshall's (1981) terms, the children tailored their references to the linguistic copresence they shared with their particular interlocutor.

On the side of comprehension, even 1-year-olds are sensitive to what is and is not linguistically co-present. Ganea and Saylor (2007) found that 15- and 18-month-olds rely on a person's prior verbal reference to an absent object to determine what the same person is speaking of a few moments later. After an adult made clear that she was searching for a particular object (e.g., a puppy), she exclaimed that she knew where “it” was and led the infant to a cabinet. Two objects—the target (puppy) and a distractor—were revealed, and the adult ambiguously asked “Can you get it for me?” Infants at both ages selected the target object, thus showing that they located the referent in the adult's prior speech. Echoing the findings on prior attentional engagement (e.g., Moll et al., 2008; Liebal et al., 2009), the infants also knew with which particular person they shared the linguistic background: When a different adult than the one who had searched articulated the request, the infants grasped objects randomly.

A further indication that infants keep track of and update records of linguistic co-presence is their appropriate use of elliptical constructions in discourse. In a study by Salomo et al. (2010), 2-year-olds were asked, “What's the agent doing now?” after watching and hearing verbal descriptions of videos showing either the same action performed on different patients (e.g., a frog feeding a duck vs. a ladybug) or different actions performed on the same patient (e.g., a frog feeding vs. washing a duck). In their answers, the children omitted reference to the patient when it remained the same and was thus given in the prior discourse. When the patient changed and was thus new, the same children made reference to it with a lexical noun. The children thus knew when null-references were and were not warranted given the discourse background. Additional evidence that 2-year-olds know which information is obligatory vs. optional in speech stems from observations of children who acquire “null-argument” languages; i.e., languages that allow the omission of subjects and objects given the appropriate discourse context (Serratrice, 2005).

Taken together, these findings clearly demonstrate that infants produce and understand gestures and speech acts against the background of their prior interactions with other persons (see Wittgenstein, 2001; Tomasello, 2008). What infuses the gestures and speech acts with meaning is the intersubjectively shared background of prior experiences. Through joint attention, infants construct a common ground (Clark, 1996) with specific other persons, and they discriminate between the dyad-specific common grounds, keeping track of what they have and have not shared with whom. In their attempts to secure reference, they naturally revert to these backgrounds, which becomes particularly obvious under conditions of potential ambiguity.

## Visual perspective-taking: no help from the background

None of the above is available in visual perspective-taking. All that is relevant here are instantaneous viewing angles and momentary spatial relations. The experiential background offers no help to solve referential ambiguity in these tests. In fact, a prerequisite that has to be met to guarantee the validity of these tests is that the candidate objects be “experientially neutral,” i.e., that target and distractor cannot be distinguished by any distinct roles they played in prior interactions. The correct response has to depend entirely on the objects' visibility (level 1) or mode of presentation (level 2 perspective-taking, see e.g., Flavell, 1992, for the distinction of level 1 vs. level 2) from a particular viewpoint. We will limit our analysis to level 1 visual perspective-taking, i.e., the ability to determine what another can and cannot see. This level of perspective-taking emerges a couple of years prior to level 2, and is structurally similar to the tasks above, which dealt with children's understanding of what others desired, witnessed, or spoke about. Level 2 is a more effortful, qualitatively distinct (Kessler and Rutherford, 2010), phylogenetically recent (human-specific) skill, that requires an explicit understanding of perspectival differences and, in the absence of autism (see Hamilton et al., 2009), emerges between 4 and 5 years.

Children first exhibit an understanding of what others can and cannot see at around 2 years of age and older. For example, when 24-month-olds witness an adult searching for something, they preferably hand her an object that is blocked from the adult's view instead of a mutually visible one (Moll and Tomasello, 2006). In a similar task by Nurmsoo and Bloom (2008), 31-month-olds also mostly selected an object that was hidden from an adult's view when he pretended to be searching for something. (One should note that there was a confound with gaze direction in this study: The adult looked straight at the visible distractor object when asking “where” the referent was, allowing children to act on a simple heuristic that people do not search for things at which they are currently looking.)

In one of several tasks administered by Masangkay et al. (1974), children between 2 and 3 correctly judged that an adult sitting across from them could not see an apple depicted on the front of a card held between them. Hughes and Donaldson (1979) found that 3-year-olds knew where to place a doll in a house so that none of several policemen at various positions could see her. In another study, 2.5−, but not 2-year-olds, granted an adult visual access to an object he desired to see by either revealing the object from behind an occluder or moving away the occluder (Lempers et al., 1977). In an “analogy task” developed by Yaniv and Shatz (1990) 3.5-year-olds were able to place a duck so that a doll perceiver saw the same part or side (e.g., its back) of the duck as another doll that looked at an identical duck.

In sum, we find that level 1 perspective-taking as demonstrated by tests using interactive methods emerges between the second and third birthday. This ability comprises percept production (enabling another to see something), percept diagnosis (judging what another sees), and percept deprivation (hiding objects from another). In Clark and Marshall's (1981 terms, it is now that children have come to understand when mutual knowledge is and is not supported by immediate physical co-presence.

However, children at this age are far from being proficient at visual perspective-taking. On the contrary, striking limitations have been identified. Under the age of 3, children are unable to hide an object from an adult by placing a barrier between her and the object (Flavell et al., 1978; McGuigan and Doherty, 2002). Two-year-olds also struggle to select appropriate referring expressions depending on what their interlocutor can see. While the children in Matthews et al.'s (2006) above-mentioned study successfully tailored their expressions to the prior discourse, they did not adjust their speech accordingly when the adult's visual access to the video was manipulated. More concretely, they did not produce more full nouns (instead of the less informative pronouns) when the adult failed to see the video compared to when he saw it. In Yaniv and Shatz's (1990) study, 3-year-olds preferably positioned the duck facing the doll, even when asked to place it so that the doll would see its back. They thus exerted a bias to generate the canonical or good—in this case the frontal—view of the object, irrespective of the instruction (see Light and Nix, 1983; more on a similar phenomenon in children's drawings below).

## A developmental lag

Taken together, these studies point at a developmental lag between social and visual perspective-taking. Infants rely on prior joint perceptual experiences including previously shared discourse at least 1 year before they take into account others' visuo-spatial relations to the things around them when they discern or establish reference. This is a significant décalage given the young age of these children. Contra Clark and Marshall (1981) and contra intuition, immediate physical co-presence does not necessarily facilitate the delineation of common ground. While it is true that physical co-presence often rightly signals that a given object figures in the common ground, the same co-presence can hampen children's ability to identify what is mutually given from what they have privileged access to as individuals. It can trick them into falsely assuming that an object they see is perceptually available to the other as well. The strong priority that is ascribed to an *ad-hoc* formation of mutual knowledge based on immediate or potential physical co-presence is thus called into question by this developmental sequence.

That the lag is real and robust becomes particularly obvious in studies in which an understanding of knowledge and ignorance is directly contrasted with visual perspective-taking. Moll et al. (2010) compared 2-year-olds' ability to detect an adult's ignorance due to absence vs. impeded vision. When the adult disengaged entirely from her interaction with the child by leaving after having shared two toys with her, the children later knew that the adult was unfamiliar with a third object that they were presented with. But when the adult remained co-present with her visual access to the third object blocked by a barrier as the child explored it, the children later acted as if the adult was familiar with this object. They failed to recognize the barrier's effect.

A very similar pattern emerged in Nurmsoo and Bloom's (2008) study. In their second experiment, 31-month-olds had no problem identifying what an adult was looking for when she had hidden one object but was absent when the other was hidden—thus making her ignorant of the second object's location. By contrast, children this age found it relatively difficult to determine what the adult searched for when he had seen neither placement but was spatially positioned so that he could not see one of the objects (Experiment 1). Similarly, and as mentioned above, while 2-year-olds in Matthews et al.'s (2006) study readily switched to more informative references when an object was not shared in prior discourse with an adult, they failed to adjust the informativeness of their speech accordingly when the referent was blocked from the adult's sight. Taken as a whole, these studies clearly show that young children can draw the knowledge-ignorance distinction before they solve otherwise identical tests that tap visual perspective-taking.

The same gap has been identified with looking-time measures as well. Again, when this method is applied, infants as young as 7 months show a sensitivity to the manipulation of perceptually induced beliefs (Kovács et al., 2010). They look longer when an agent behaves in a way that is inconsistent with what she witnessed earlier than when her behavior matches her prior observations (someone looks for something where she last saw it). In contrast, the youngest age for which level 1 visual perspective-taking has been documented with the looking-time technique is 13–16 months (Luo and Baillargeon, 2007; Sodian et al., 2007; Luo and Beck, 2010). For example, when 13-month-olds repeatedly see an adult reaching for one of two toys, they form an expectation that he will keep doing so—as evidenced by longer looks when he suddenly reaches for the previously ignored toy. But they only form this expectation when the agent is able to see the alternative object, and thus disprefers it. No extended looks were shown when the non-chosen toy was blocked from the agent's view, and so simply unseen.

Further indication that 13-month-olds have rudimentary skills in visual perspective-taking stems from a study that is purported to test false belief comprehension (Surian et al., 2007). In this looking-time experiment, a caterpillar's knowledge of his preferred object's location was manipulated by the presence/absence of a barrier impeding the caterpillar's, but not the child's, vision of the object. The authors do not interpret their results in terms of visual perspective-taking. But partly because looking-time measures involve no “task” (the child is not asked or prompted to respond to anything in particular), it remains open which aspect infants mainly reacted to or found harder to process: realizing the barrier's defeating effect on the agent's vision, or keeping track of what he did and did not witness.

In either case, the same developmental lag that is found with interactive response methods becomes manifest when looking times are applied, albeit at a younger age—reflecting the reduced task affordances of this method. The fact that the developmental order pervades different research methods shows its robustness. But it has to be emphasized that the lag is limited to level 1 visual perspective-taking and its corresponding counterparts in social perspective-taking. A more synchronous pattern is found at level 2, which affords an explicit knowledge of the possibility of alternative, and potentially false, views. This knowledge, which spans across visual and social perspectives alike, is formed between 4 and 5 years—supporting the idea of a common cognitive thread that runs through various perspective problems (see Perner et al., 2003; Moll and Meltzoff, 2012). The gap that calls for an explanation thus only exists in the early beginnings of perspective-taking, before a more abstract and uniform understanding of perspectives develops in late preschool.

The pressing question then is how the counterintuitive sequence of visual perspective-taking preceding social perspective-taking observed in the early years can be explained. We will make a first explanatory attempt by addressing the more specific question of why visual perspective-taking might pose a particular challenge (see Moll and Meltzoff, 2012).

### Shared perceptual spaces

We argue that young children have a proclivity to treat social interaction as a sufficient condition for shared perceptual availability: “When you and I are co-present and engaged, you should be able to perceive what I perceive.” An impression of a shared perceptual space is induced, and only later overcome once children learn more about and attend more to the specific defeating conditions of perception, such as a blocked line of sight.

Support for the idea that co-presence and social engagement create an illusion of shared perception comes from experimental data with both children and adults. Glucksberg and Krauss (1967) report that preschoolers produced iconic gestures and used demonstratives (“It goes like this!”) to describe objects to their conversational partner who sat across from an occluder. The work of Keysar and colleagues shows that even adults have a prepotent tendency to assume that others around them share their perceptual access to objects, even when this is not true (e.g., Keysar et al., 2003; Epley et al., 2004; Keysar, 2007). Consistent with what we know about children, adults are biased to over-rather than underestimate what the other sees or knows (Keysar and Henly, 2002; see also Bernstein et al., 2007). Interestingly, the thicker or richer the common ground shared by two people, the more likely they are to overrate the success of their communicative attempts (Wu and Keysar, 2007). The more that is shared, the less prepared one is to identify when something is not shared. A vast overlap in what is perceptually accessible weakens the alertness to check if a particular object is mutually given or not. In support of this, it was found that people communicate less informatively to a concrete other person who “co-inhabits” their perceptual space than to a merely imagined interlocutor (Schober, 1993). This is much in line with our developmental finding that corporeal co-presence, and thus a high overlap in what can potentially be turned into an object of shared attention, hampens young children's ability to detect others' ignorance (Moll et al., 2010).

This overestimation effect also helps to explain young children's notoriously poor perspective-taking skills when speaking on the phone. It has long been known that children use manual gestures, demonstratives, and non-specific references during phone conversations (Bordeaux and Willbrand, 1987; Warren and Tate, 1992)—indicating that they are unaware of the fact that they and the things around them cannot be seen. In our interpretation, the shared discourse elicits the false impression of a generally shared perceptual space that spans across different sense modalities, including vision. That is, verbally established co-presence leads to the illusory impression of shared visual perception.

This idea, however, is called into question by experiments suggesting that others' viewpoints make their way into our considerations effortlessly and automatically (Qureshi et al., 2010). When asked how many items they see in a visual array, adults and school-age children are slower and less accurate in their judgments if their visual input mismatches that of another agent who is part of the scene they watch (Samson et al., 2010; Surtees and Apperly, 2012; see also Surtees et al., 2012). Two things can be said to reconcile these findings with our overestimation thesis. Firstly, it is conceivable that once level 1 visual perspective-taking has been practiced for years, it becomes “second nature” or automated. Secondly, the participants' situation differs drastically between the studies. In those studies supporting the overestimation thesis, the child interacts with the other directly, which might let the perspectival differences between them dissolve “in the heat of the moment.” In the tasks suggesting automatic perspective-taking, participants have a contemplative, theoretical distance to the other, who figures in the array like an object. This theoretical distance could highlight the other's position in relation to the remaining items in the scene. The two sets of findings thus do not necessarily contradict each other.

### A glance at early picture-making

It was speculated that before children's perception is corrupted by language and thought, they ought to see the world with innocent, i.e., objective eyes (see Matisse, 1953) and even master perfectly the art of drawing in linear perspective (Bühler, 1930; Sully, 1895). But of course, by the time children have the motor skills and motivation to depict objects and events, they have long been language- and concept-using beings who have passed any hypothetical phase of innocent vision (see Costall, 1997, 2001).

When children begin to draw figuratively, they do not faithfully translate three-dimensional objects onto two-dimensional picture planes. They show no intention to depict things exactly the way they appear to them from one fixed point of observation. Drawing does not serve the goal of imitating visual experiences. As a famous dictum says, children “draw what they know, not what they see” (but see Arnheim, 1974, p. 164, for rightly criticizing the false opposition of seeing and knowing that is employed here)—exhibiting a style dubbed “intellectual realism” (Luquet, 1927). They include aspects and elements in their pictures that cannot be seen from their present perspective and may not be visible from any particular, single viewpoint. They create a “good” or ideal view of objects by depicting features they consider relevant or important and omitting what is irrelevant. The goal is not to produce a correct perspectival reconstruction but to show objects in their typical form and thus to capture their constitutive or essential features. For example, a cup will be depicted in canonical fashion with a handle on its side (ideal for grasping, see Cox, 1991). Likewise, humans are shown in their canonical frontal view with a face including two eyes (ideal for social interaction), whereas trunk, nose and other parts might be left out (Cox, 1997).

Also, young children mostly produce images spontaneously from memory and imagination (Golomb, 2004). When presented with a model to guide their drawing activity, they rarely look up to see what the object exactly looks like. The model serves as a source of inspiration—it provides a theme or motif and is relevant insofar it exemplifies a generic object (Luquet, 1927), but it is not adhered to as an original that ought to be replicated. Again, what this indicates is that children do not intend but fail to draw from a fixed perspective.

In his essay “Perspective as symbolic form,” Panofsky (1927) pointed out that a faithful reconstruction of what is seen from a particular viewpoint affords a severe abstraction from the content of experience. In his own words, it is a modern technique that rests on a motivation to strip away the experiential or “given” space and substitute it with a systematic, purely visual space. An individualistic and somewhat arbitrary factor thereby gets introduced, because one commits to showing the scene from a single, static point of observation. Any ordering according to what is regarded important or relevant has to make way for a strictly geometric ordering.

With this held in mind, it becomes much less puzzling why an awareness of visual perspectives emerges rather late—not just in history, but in ontogeny as well (see Gablik, 1977, for parallels between the history and genetic development of visual art). Though children at age 5 and older can be induced to draw what they see, it is not before 7 or 8 years that they spontaneously create view-dependent images (Davis, 1983; Cox, 1991). Even at this age, their advances are such that they acknowledge partial occlusion and draw only what is visible (e.g., the correct number of faces of a cube), but they still do not depict the visible parts precisely in the way they appear (e.g., with lines converging in a vanishing point; Bremner and Batten, 1991; Cox, 1991).

What is of primary importance to children is to share the world of those around them. Precisely how this shared world presents itself from one specific vantage point is secondary and does not become thematic in the very early stages. First and foremost, drawing serves to “make sense of the world” (Arnheim, 1969, p. 257)—and this is true ontogenetically as well. Young children draw to narrate events and give “shape and order” (Cox, 2005) to their experiences. We want to go further and argue that picture-making primarily serves to make sense of the social world, as one of the first and most frequent motifs is the human figure (Maitland, 1895; Lark-Horovitz et al., 1939; see Cox, 1993, for an overview). But not just the themes or motifs are social; so is the process of drawing. It is an activity that is typically shown in the presence of another to whom the child narrates as she draws, and for whom she might create the picture as a gift. The graphic product in itself can hardly be interpreted without the accompanying speech in which children reconstruct their experiences and reveal what they intend to draw (Cox, 2005).

In either case, we find that the relatively late onset of taking others' visual perspectives is paralleled by a late emergence of the use of perspective in drawings. Young children's pictures document their inattention to specific visual perspectives. Just like there was no motivation to graphically capture objects from specific, transient viewpoints in the early history of visual art (Panofsky, 1927), so do children show no interest in representing things precisely the way they happen to see them. They ignore the contingent ways in which things appear momentarily for the sake of capturing what belongs to an object more generally. This also becomes manifest in perceptual self-reports. When preschoolers are asked to indicate how they perceive a visual array by choosing from among a set of different pictures, they often judge incorrectly and select a picture showing the ideal rather than their own view (Liben and Belknap, 1981; Light and Nix, 1983). The upshot is that children's drawings are one of several pieces of converging evidence that young children pay little attention to differences in visual perspective. Others are their faulty perceptual reports, their behavior during phone conversations, and, as we have seen, profound struggles with visual perspective-taking—neither of which require graphic skills.

## Concluding remarks

Humans are extraordinarily *relational* and interdependent beings (MacMurray, 1961). They are adapted to rely on and cooperate with others in a way that is unparalleled in the animal kingdom (Gintis et al., 2003). Especially in the early beginnings, a human individual is entirely dependent on others' care, attention, and sharing of knowledge (Csibra and Gergely, 2011). What is crucial at this early stage is that the child comes to share the world of those around her. She accomplishes this by jointly attending to things with others. It is in these bouts of joint attention that the child learns about objects: their gestalts, functions, and labels etc. Importantly, these are perspective-invariant properties. The focus lies on the object and its qualities, not on the different perspectives from which each co-attender perceives it (Campbell, 2012; Moll and Meltzoff, 2012; Seemann, 2012). Only once it can be taken for granted that we attend to the same thing, is there room, in a second step, to “objectify” the different viewpoints from which each of us perceives the object. As Campbell (2012, p. 428) puts it, “The point is that a grasp of the different perspectives from which a thing may be experienced should not be allowed to take on a life of its own; this grasp of the different perspectives from which a thing may be experienced is always grounded in a prior knowledge of which thing is in question.”

But this merely seems to explain why joint attention precedes knowledge of perspectives, not why children engage in social perspective-taking before visual perspective-taking. However, we think that these two things are related. In joint attention, one's knowledge of the object becomes mutually transparent and so does the expression of one's attitude toward it. While the focus of joint attention is the object itself, it simultaneously informs us of the other's knowledge of it as well as her take on it. Joint attention thus directly supports the forms of social perspective-taking discussed in this article, which are critical for cooperative communication and other forms of collaborative activities.

The visuo-spatial positions of the co-attenders, in contrast, remain entirely in the background. Firstly, the viewing angles involved usually bear no significance with regard to the object, its qualities, or the other's attitude toward it. Secondly, given the dynamic character of joint attention, these perspectives rarely remain constant but tend to fluctuate over the course of exploration, as the object gets manipulated and/or the spatial positions changed. Joint attention thus directly paves the way to early forms of social, but not visual perspective-taking.

The picture looks very different for non-human primates that possess simple forms of visual perspective-taking. Chimpanzees have been shown to preferably approach food that is blocked from a dominant individual's sight (Hare et al., 2000), and to seek out locations and motion paths that hide their bodies from competitors (Whiten and Byrne, 1988; Hare et al., 2006). These behaviors are advantageous in potentially antagonistic and risky encounters with conspecifics or predators (Hare and Tomasello, 2004). They are evolutionarily adaptive for animals that are yoked much tighter into the here and now than humans and do not engage in shared intentionality and cooperation. We think that joint attention and cooperation bridge spatial distances between self and other and thus privilege social over visual perspective-taking. The competitive and individualistic mode of operating found in non-human primates, in contrast, makes an awareness of the visibility of resources and one's own body to others critical for survival.

Generally, visuo-spatial perspective-taking is seen as the most basic and embodied form of perspective-taking, that is expected to subserve and function as a model for more mental or higher-cognitive forms, such as imagining how others feel or think about a certain situation (as is also suggested by spatial metaphors such as “putting oneself in another's position/shoes,” see Kessler and Thomson, 2010). The genetic primacy of social over visual perspective-taking that we argued and provided empirical support for is at odds with this idea of visual perspective-taking as the cradle for other kinds of perspective-taking. Being aware of and responsive to others' literal viewpoints can certainly be key in social interaction. To communicate effectively we often have to adjust our speech and non-verbal behavior according to what the other sees or how he sees things—e.g., when we direct him to an object outside of his visual field or ask him to move his left shoulder that is on our right as we stand facing him. But getting a grip on others' visual perspectives takes time ontogenetically, and is not the first skill of its type to emerge.

We tried to show in this article that before children come to know what is seen from which particular viewpoint, they not only bridge perspectival differences in acts of joint attention and deictic reference by the age of 9–12 months, allowing them to create a common ground of shared experience with others. They also readily track and update what others have witnessed, done, and said. This knowledge is foundational for effective communication and other forms of cooperation, as it constitutes the background against which gestures and speech acts are understood and produced. We cited empirical evidence that children develop an awareness of visual perspectives somewhat later. This is not only suggested by the relatively late onset of visual perspective-taking, but is also reflected in children's aperspectival drawings and false perceptual judgments. In our attempt to explain the counterintuitive sequence from social to visual perspective-taking we highlighted the primary importance of forming experiential backgrounds with others for the sake of communication and cooperation. If the developmental trajectory that we traced is informative with regard to the relation between visual and social perspective-taking in cognitively mature human beings remains an open question.

### Conflict of interest statement

The authors declare that the research was conducted in the absence of any commercial or financial relationships that could be construed as a potential conflict of interest.
